# Site‐directed mutagenesis of Serine‐72 reveals the location of the fructose 6‐phosphate regulatory site of the *Agrobacterium tumefaciens* ADP‐glucose pyrophosphorylase

**DOI:** 10.1002/pro.4376

**Published:** 2022-06-26

**Authors:** Mashael A. Alghamdi, Rania A. Hussien, Yuanzhang Zheng, Hiral P. Patel, Matías D. Asencion Diez, Alberto A. Iglesias, Dali Liu, Miguel A. Ballicora

**Affiliations:** ^1^ Department of Chemistry and Biochemistry Loyola University Chicago Chicago Illinois; ^2^ Department of Chemistry Imam Mohammad Ibn Saud Islamic University (IMSIU) Riyadh Saudi Arabia; ^3^ Department of Chemistry Al Baha University Al Baha Saudi Arabia; ^4^ Instituto de Agrobiotecnología del Litoral (UNL‐CONICET) FBCB Paraje “El Pozo”, CCT‐Santa Fe Santa Fe Argentina

**Keywords:** ADP‐glucose pyrophosphorylase, allosteric regulation, enzyme engineering, glycogen biosynthesis

## Abstract

The allosteric regulation of ADP–glucose pyrophosphorylase is critical for the biosynthesis of glycogen in bacteria and starch in plants. The enzyme from *Agrobacterium tumefaciens* is activated by fructose 6‐phosphate (Fru6P) and pyruvate (Pyr). The Pyr site has been recently found, but the site where Fru6P binds has remained unknown. We hypothesize that a sulfate ion previously found in the crystal structure reveals a part of the regulatory site mimicking the presence of the phosphoryl moiety of the activator Fru6P. Ser72 interacts with this sulfate ion and, if the hypothesis is correct, Ser72 would affect the interaction with Fru6P and activation of the enzyme. Here, we report structural, binding, and kinetic analysis of Ser72 mutants of the *A. tumefaciens* ADP‐glucose pyrophosphorylase. By X‐ray crystallography, we found that when Ser72 was replaced by Asp or Glu side chain carboxylates protruded into the sulfate‐binding pocket. They would present a strong steric and electrostatic hindrance to the phosphoryl moiety of Fru6P, while being remote from the Pyr site. In agreement, we found that Fru6P could not activate or bind to S72E or S72D mutants, whereas Pyr was still an effective activator. These mutants also blocked the binding of the inhibitor AMP. This could potentially have biotechnological importance in obtaining enzyme forms insensitive to inhibition. Other mutations in this position (Ala, Cys, and Trp) confirmed the importance of Ser72 in regulation. We propose that the ADP‐glucose pyrophosphorylase from *A. tumefaciens* have two distinct sites for Fru6P and Pyr working in tandem to regulate glycogen biosynthesis.

## Abstract 2

PDB Code(s): 6V9A, 6V96 and 6V99;

**Abbreviations**
ADP‐Glc PPase: ADP‐glucose pyrophosphorylase
ADP‐Glc: ADP‐glucose
AM: ammonium molybdate
AMP: adenosine monophosphate
CDW: cell dry weight
FBP: fructose‐1,6‐bisphosphate
Fru6P: fructose‐6‐phosphate
Glc1P: glucose‐1‐phosphate
IPTG: isopropyl β‐d‐1‐thiogalactopyranoside
LB: Luria broth
MG: Malachite green oxalate salt
PPi: inorganic pyrophosphate
Pyr: pyruvate
T20: tween‐20

## 1 INTRODUCTION

ADP‐glucose (ADP‐Glc) is the molecule acting as the donor of glycosyl‐moieties for glycogen and starch synthesis in bacteria and plants, respectively.^1^, ^2^ The production of this metabolite is catalyzed by the enzyme ADP‐glucose pyrophosphorylase (ADP‐Glc PPase, EC 2.7.7.27). ADP‐Glc PPase catalyzes the rate‐limiting step in the process of synthesizing the storage polysaccharide in bacteria and plants, by converting ATP and glucose‐1‐phosphate (Glc1P), in the presence of a divalent cation (Mg^2+^) into ADP‐Glc along with inorganic pyrophosphate (PPi).^3^ Subsequently, the glycogen/starch synthase and branching enzyme catalyze the reactions required for α‐1,6‐glucan chain branching to build up the polysaccharide.^4^, ^5^

The ADP‐Glc PPase is allosterically regulated by key intermediates of the major carbon assimilation route in different organisms. These metabolites regulate the enzyme activity according to the condition of the cells, as the activators represent signals of the fed state while the inhibitors represent the starvation situation.^1^, ^2^ ADP‐Glc PPases from *Escherichia coli* and *Agrobacterium tumefaciens* have been relatively well studied and they exhibit differences with respect to specificity for allosteric regulators.^1^, ^6^, ^7^ Thus, in *E. coli* (and other enteric bacteria), which uses the Embden‐Meyerhoff‐Parnas as the main glycolytic pathway, the enzyme has fructose‐1,6‐bisphosphate (FBP) and AMP as major activator and inhibitor, respectively. Distinctively, in *A. tumefaciens*, having the Entner–Doudoroff glycolytic pathway, the ADP‐Glc PPase is activated by fructose‐6‐phosphate (Fru6P) and pyruvate (Pyr) (with no major effect of FBP) and AMP is the main inhibitor.^1^, ^6^

All the ADP‐Glc PPases described so far are tetrameric enzymes with a molecular mass of ~200 kDa.^1^, ^2^ Different studies were useful to identify functional enzyme domains involved in regulation, including those participating in propagation of the signal triggered by the allosteric activator.^6^, ^8^, ^9^, ^10^, ^11^, ^12^, ^13^, ^14^ At present, crystal structures of ADP‐Glc PPase from *A. tumefaciens*, *E. coli*, and potato tuber have been reported.^15^, ^16^, ^17^, ^18^, ^19^ In one of these recent structural works, we found a Pyr activator site in the *A. tumefaciens* enzyme,^18^ but the site for the binding of Fru6P (the other main activator for this protein) has not been observed thus far. Based on kinetic and structural information, Fru6P would not fit in the Pyr site leading to the hypothesis that this enzyme has another distinct site for Fru6P.^18^, ^20^

Previous studies determined that the allosteric regulation of the ADP‐Glc PPase from *A. tumefaciens* involves residues located at both the N‐ and the C‐terminus in the protein.^6^ It was shown that Arg32, Arg33, and Arg45 are N‐terminal residues with an important role in the activation process.^14^ Mutation of Arg32 significantly decreased the enzyme affinity toward Fru6P. Arg32 is homologous to Lys39 in the *E. coli* enzyme, which is involved in the binding of the activator FBP.^21^ Mutation of Arg33 and Arg45, which are conserved residues in different species, produced pre‐activated enzymes with loss of sensitivity to Fru6P.^14^ In addition, Arg33 and Arg45 interact with a sulfate ion present in the crystal structure in the cleft between N‐ and C‐terminal domains.^17^, ^18^ Homologous residues in the *E. coli* enzyme (Arg40 and Arg52) have a significant role in the activation by FBP,^11^ highlighting their regulatory importance in the family.

Some of the residues surrounding that sulfate in the cleft between N‐ and C‐terminal domains seem to have a regulatory role in the *A. tumefaciens* ADP‐Glc PPase. For example, His379 locates at the C‐terminal side and, when mutated, the specificity for activators FBP and Fru6P was altered.^17^ We also observed that Ser72 is interacting with the sulfate ion,^18^ although the role of this residue in the enzyme allosteric regulation has not been revealed. We hypothesize that this sulfate occupies the site where the phosphate moiety of the activator Fru6P binds. If such a model is correct, all the residues interacting with the sulfate in the crystal structure should participate in the binding of Fru6P. In this framework, we studied several mutations of Ser72 with the idea of altering the binding interaction. Some mutants lacked the ability to form a hydrogen bond and others were predicted to sterically block the putative binding of Fru6P. In addition, we crystallized mutants S72E and S72D in the presence and absence of sulfate to correlate structural information with our kinetic results.

## 2 RESULTS

**FIGURE 1:**
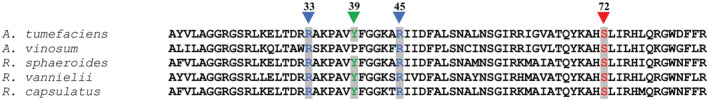
Sequence alignment of ADP‐Glc PPase known to be activated by Fru6P. Sequences are from *Agrobacterium tumefaciens*, *A. vinosum*, *Rhodobacter sphaeroides*, *R. vannielii*, and *R. capsulatus*. Highlighting Ser72, Arg33, Arg45, and Tyr39 positions, which might be involved in the binding of Fru6P and AMP. The sequence alignment processed as described in Section 4

The potential role of the hypothesized regulation cleft in the *A. tumefaciens* ADP‐Glc PPase has been studied by substituting Arg32, Arg33, and Arg45, which are located nearby Ser72. This residue, as well as Arg33 and Arg45, interact with oxygen atoms of a sulfate ion observed in the crystal structure.^17^, ^18^ The hypothesis that this particular sulfate occupies the place of the phosphoryl moiety of the Fru6P activator is supported by the fact that sulfate behaves like a competitive inhibitor of Fru6P.^17^ In addition, Ser72 is conserved in all ADP‐Glc PPases that are activated by Fru6P (Figure 1). Taken together, Ser72 is a reasonable candidate to be investigated in the context of allosteric regulation of the enzyme. This hypothesis can be tested by removing the interactions with the effector molecules introducing mutations in this position that block the binding of the activator.

### 2.1 Ser72 substitution

We replaced the Ser72 in the *A. tumefaciens* ADP‐Glc PPase with five different residues. One of the replacements was Ala, which removes possible interactions by the Ser hydroxyl group. We also replaced Ser with Asp and Glu, which may block the binding of a negative phosphate moiety. A thiol of Cys mutation would have different hydrogen bonding properties from the hydroxyl group. The introduction of Trp, as a bulky amino acid, would provide data regarding steric hindrances with the activator.

### 2.2 Kinetic characterization

#### 2.2.1 Effects of mutations on Fru6P regulation

**FIGURE 2:**
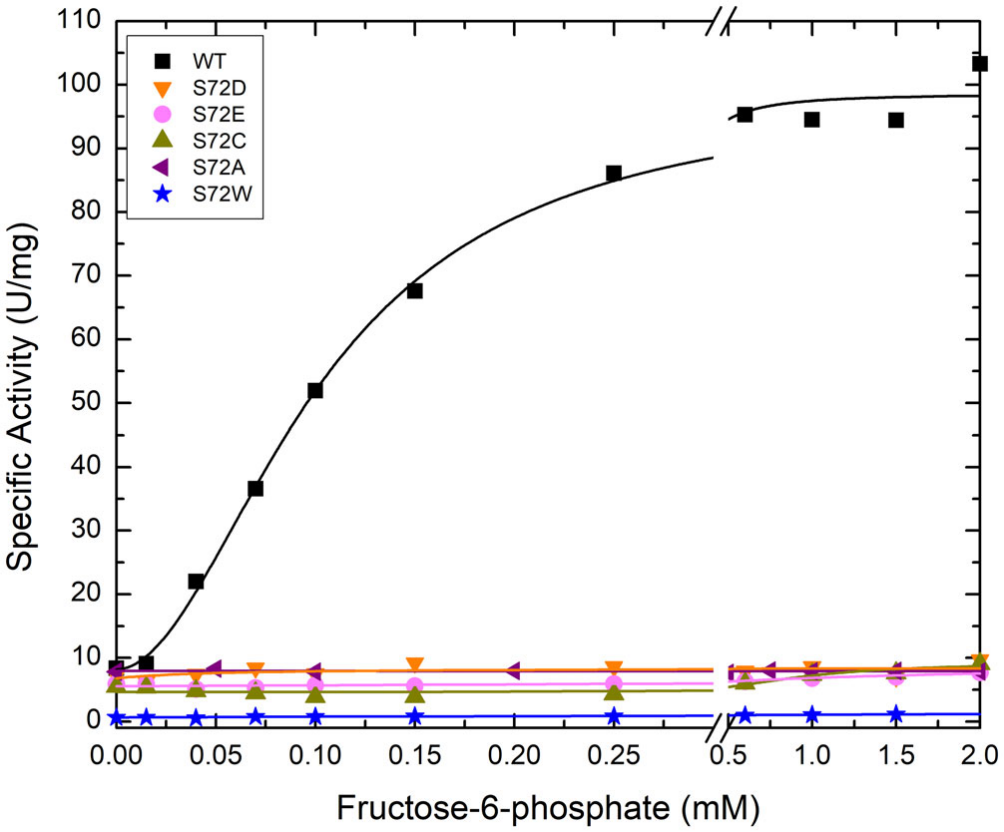
Activator (Fru6P) saturation curves for the *A. tumefaciens* ADP‐Glc PPase WT and mutants. The enzymes were assayed by the production of PPi as indicated in Section 4

The regulatory effects of Fru6P on the wild‐type ADP‐Glc PPase and several site‐directed mutants are illustrated in Figure 2. It was evident that the mutant enzymes, compared to the WT, were insensitive to the addition of Fru6P. The activation of the WT by Fru6P increased the activity ~12‐fold (98.5 U/mg), whereas the mutants showed no significant response to Fru6P even at higher concentrations (Table S1). The maximum activity (in assays in the presence of Fru6P) for S72A, S72D, S72E, S72C, and S72W was one order of magnitude lower. Except for S72W (exhibiting a modest two‐fold activation), all the mutant enzymes displayed similar activities with the WT in the absence of the activator. This implied that the mutations neither significantly altered the nonactivated basal activity nor disrupted the structure (they just were unresponsive to the activation). The activity of the S72W mutant compared to the wild type was about ~6‐fold lower (Table S1), which could be explained by the introduction of a bulky side chain. For that reason, analysis of the other mutations is more relevant, but the S72W data are still included for the sake of completeness.

#### 2.2.2 Effects of mutations on Pyr regulation

**FIGURE 3:**
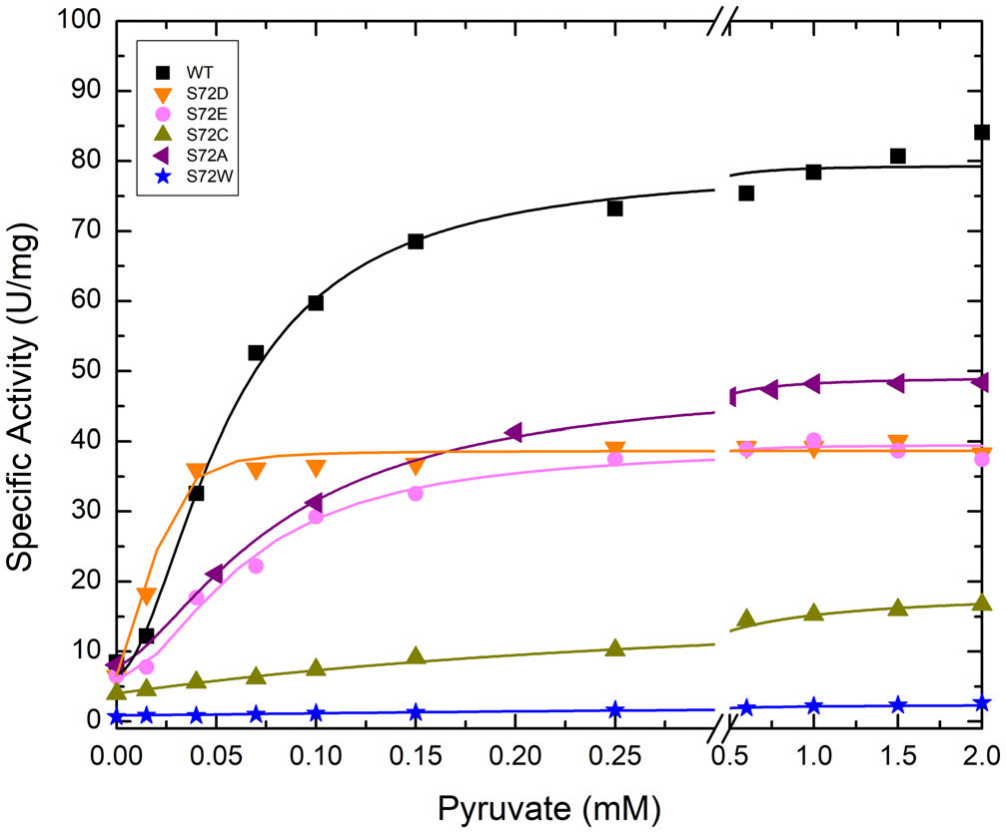
Activator (Pyr) saturation curves for the *Agrobacterium tumefaciens* ADP‐Glc PPase WT and the mutants. The enzymes were assayed by the production of PPi as indicated in Section 4

The Pyr saturation curves for wild type and mutant forms of *A. tumefaciens* ADP‐Glc PPase were analyzed (Figure 3). As opposed to the results with Fru6P, there were significant activation with Pyr (except S72W). The Pyr apparent affinities for S72A, S72D, and S72E (18–83 μM) were not very different from the WT (57 μM, Table S2). In addition, the activation fold by Pyr in these mutants was only slightly lower than the WT. For example, when we introduced an Ala (S72A), the mutant was activated ~6‐fold by Pyr, whereas WT was activated ~10‐fold (Table S2). The *V*_m_ of S72A was about 49.1 U/mg in the presence of 2 mM Pyr, which is 62% of the WT activity (Table S2).

#### 2.2.3 Effects of mutations on AMP regulation

**FIGURE 4:**
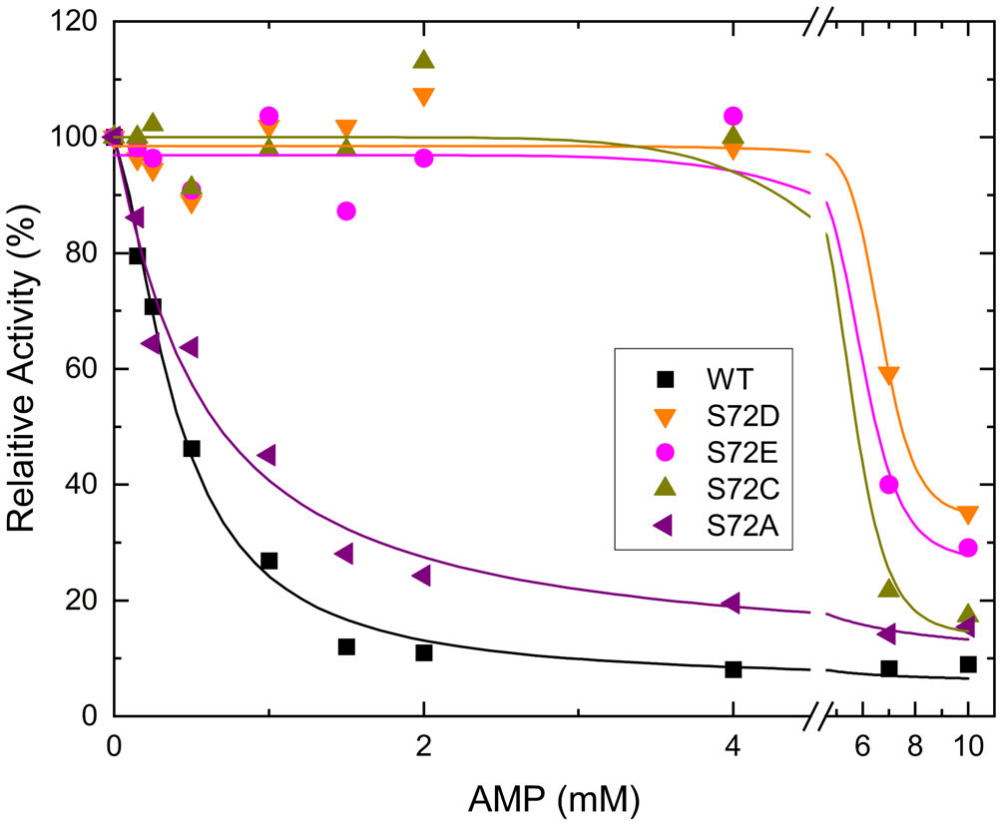
The inhibitor (AMP) saturation curves for the *Agrobacterium tumefaciens* ADP‐Glc PPase WT and the mutants. The enzymes were assayed by the production of PPi as indicated in Section 4 in presence of 0.1 mM Fru6P. The 100% of the relative activity is normalized to the one in the absence of AMP (*V*_0_). The absolute values of *V*_0_ are in Table S3

Except for S72A, the inhibition of the mutant ADP‐Glc PPases by AMP was severely affected (Figure 4). The rest of the mutants showed no detectable response to the inhibition by AMP up to ~4 mM. At higher concentrations, there was a distinct and very sigmoidal inhibition by AMP (Figure 4), but at those concentrations, we are not certain whether the effect is allosteric. For instance, AMP could be interfering by competing with ATP in the active site. On the contrary, the *I*_0.5_ of WT was 0.4 mM (Table S3). The calculation of *V*_∞_/*V*_0_ illustrates that mutants S72D, S72E, S72C, and S72W all retain a significant fraction (>13.9%) of activity at high concentrations of AMP (Table S3; Figure 4). The WT enzyme also retained some activity, but to a lesser extent (~6%) (Table S3).

#### 2.2.4 Effects of mutations on ATP substrate kinetics

**FIGURE 5:**
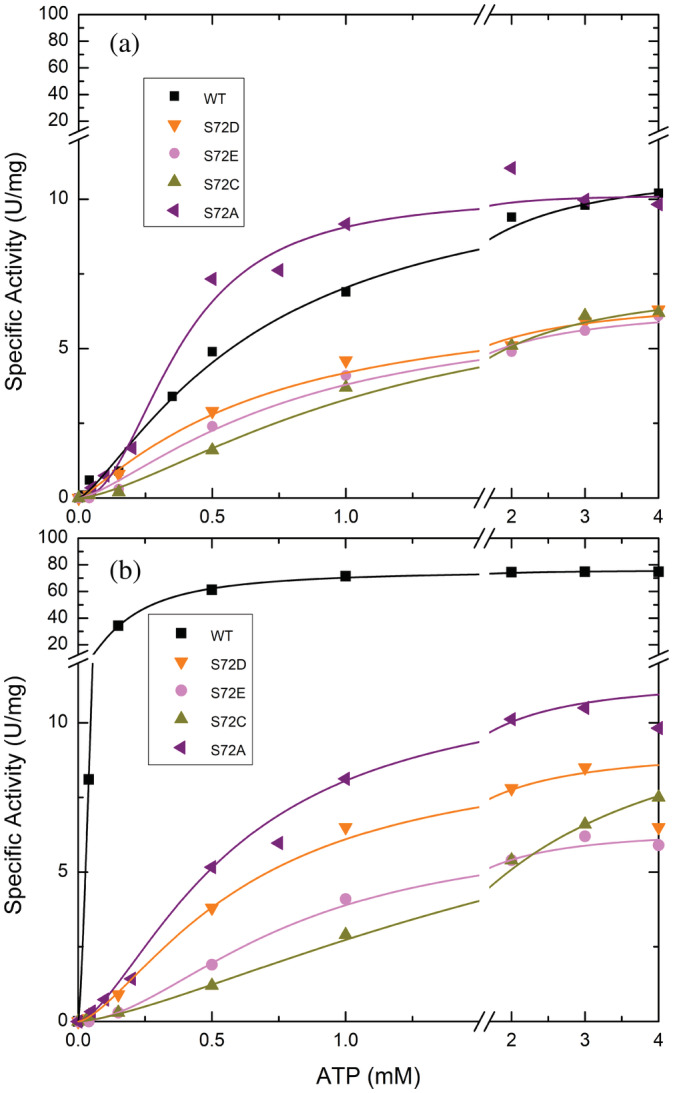
Effect of Fru6P on the ATP saturation curves of the WT and *Agrobacterium tumefaciens* ADP‐Glc PPase mutants. Panel (b) was conducted in the presence of 1.5 mM Fru6P and Panel (a) in absence. Enzymes were assayed as described in Section 4

It has been reported that the activator Fru6P can increase the *V*_m_ and the apparent affinity (decreasing the *S*_0.5_) of ATP in the *A. tumefaciens* ADP‐Glc PPase.^14^ Because of this interplay between the activator and the substrate ATP, we analyzed the ATP saturation curves of the mutants. We confirmed this behavior for the WT because the *S*_0.5_ for ATP substrate decreased ~4‐fold in the presence of 1.5 mM of the activator Fru6P (Table S4; Figure 5a,b). Conversely, none of the mutants exhibited any increase in the ATP affinity (Table S4; Figure 5a,b).

### 2.3 Thermal shift assays

**FIGURE 6:**
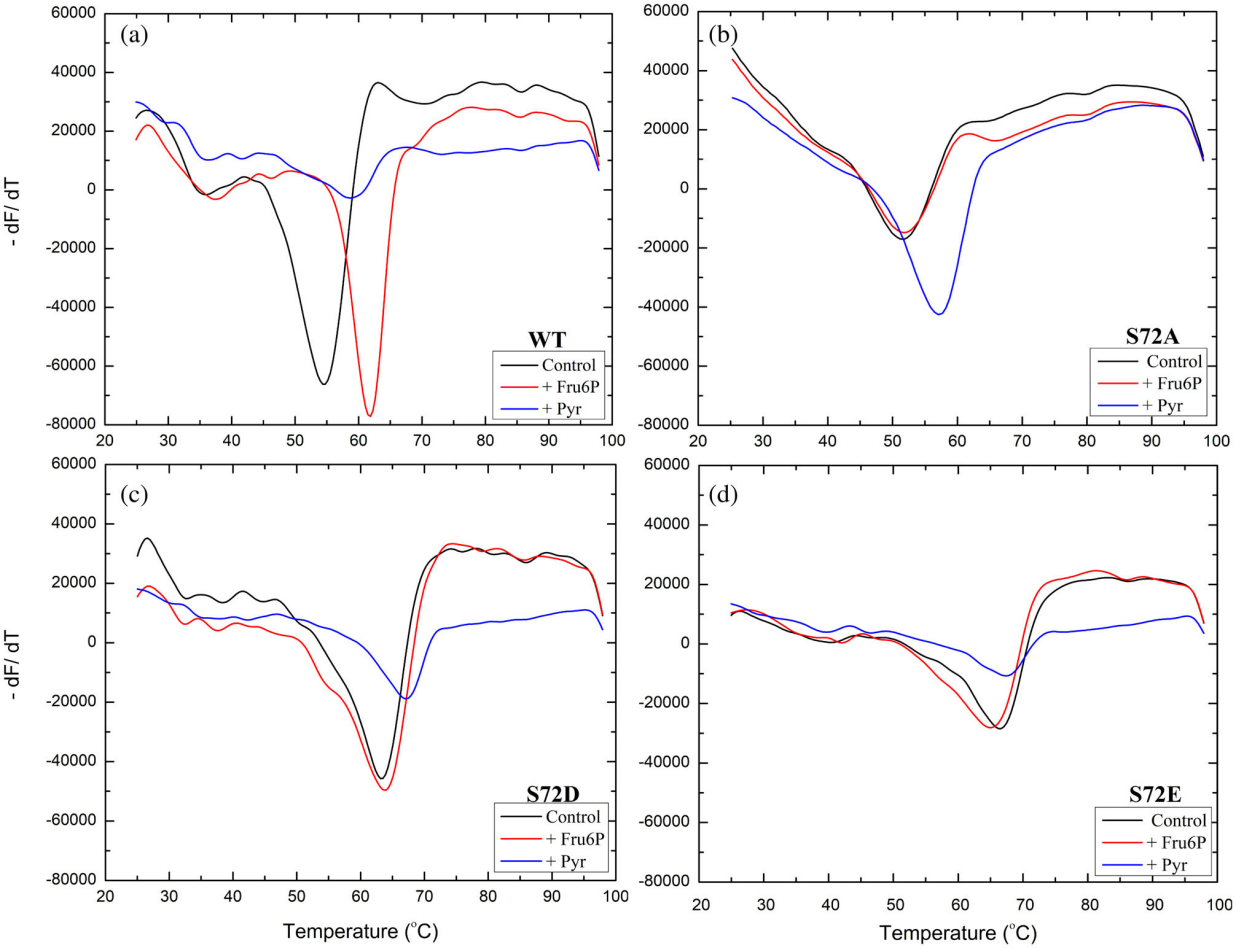
Effect of activators in on the thermal stability of the *Agrobacterium tumefaciens* ADP‐Glc PPase. Thermal shift assays for the WT and the mutants in presence and absence (control) of Fru6P (1 mM) and Pyr (1 mM) were performed as described in Section 4, with the additional presence of 1 mM ATP and 1 mM MgCl_2_ in all wells. We depicted in panels (a–d), the wild type and mutants S72A, S72D, and S72E, respectively

The WT *A. tumefaciens* ADP‐Glc PPase showed a significant shift of the *T*_m_ from 54.8°C (control) to 61.8 and 57.5°C with the addition of 1 mM Fru6P or 1 mM Pyr, respectively (Figure 6; Table S5). Therefore, the method detects that activators bind to the enzyme and stabilize it. On the contrary, adding Fru6P did not seem to stabilize S72A, S72D, and S72E (Figure 6). However, the presence of Pyr caused a small (but still noticeable) shift in the mutant S72E, whereas it significantly shifted the temperature for S72A, S72D (Figure 6). The same effect was observed in the absence of the substrate in the reaction mixture (Figure S1; Table S6). The *T*_m_ did not increase after the addition of Fru6P, but it did with the addition of Pyr (Figure S1; Table S6). The temperature shifts by Pyr indicated that these mutations did not prevent binding. This is in good agreement with the activation observed (Figure 3) and the location of the Pyr‐binding site recently found.^18^ The mutants S72C and S72W gave thermal assay patterns with a shallower slope in the presence and absence of activators. This suggests that they were already quite unstable, and *T*_m_ could not be measured accurately. Interestingly, the presence of Asp and Glu at position 72 strongly stabilized the enzyme even in the absence of activators and thus the melting temperature jumped from 54.8°C to 63.5°C and 66.1°C for S72D and S72E, respectively. This agrees with the fact that this region is highly positive with several Arg residues in the interface between the N‐ and C‐terminal domains. The addition of a negative charge by mutagenesis could partially neutralize this area, reduce the repulsion of positive charges, and consequently stabilize the folded structure of the enzyme in absence of activator. In agreement with this concept, sulfate (which can bind in this area^18^) also stabilize the enzyme. The addition of 10 mM lithium sulfate increased the *T*_m_ by 2.3°C (not shown).

### 2.4 Crystal structure

**FIGURE 7:**
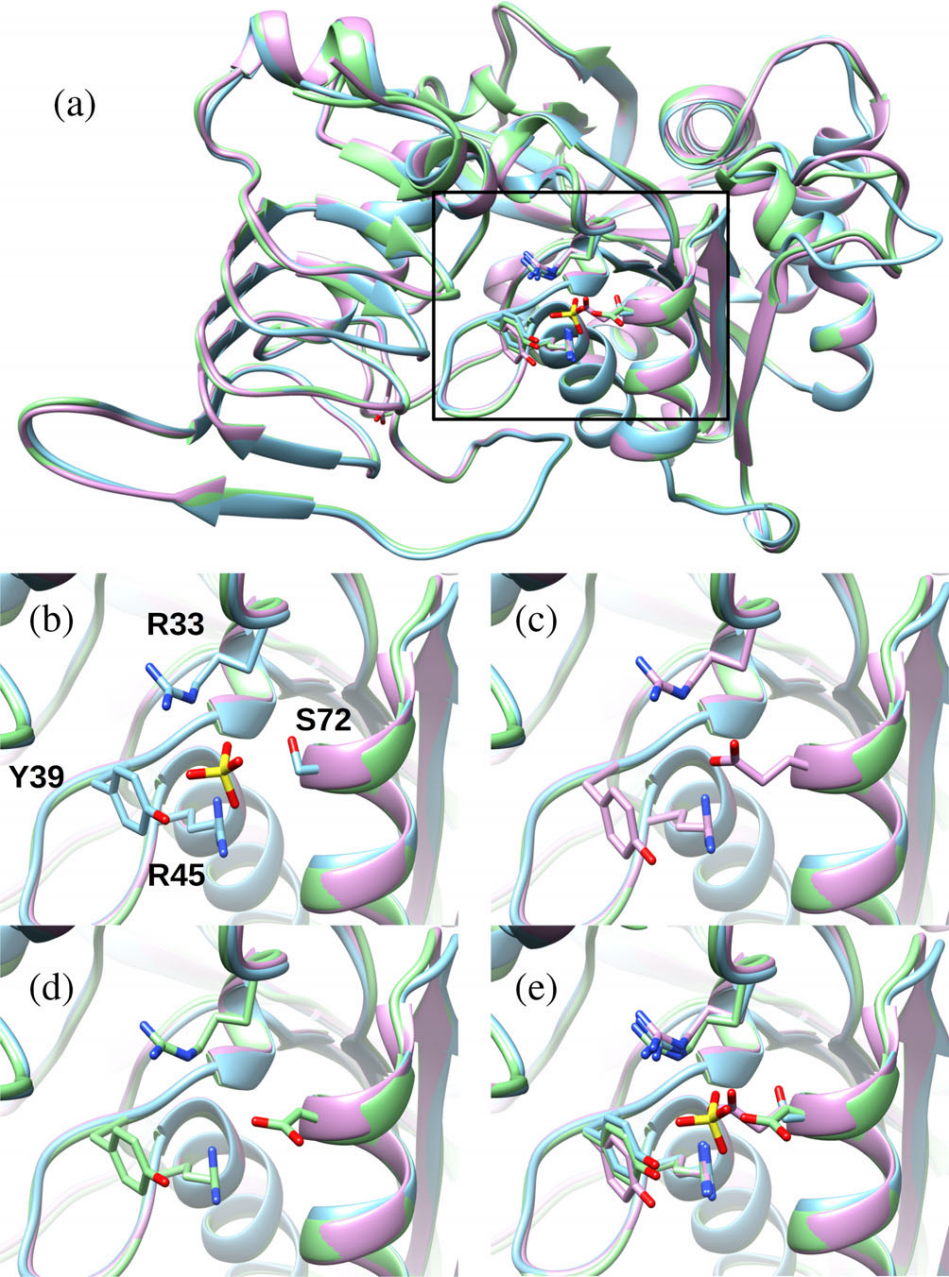
Comparison of the crystal structure of the wild‐type ADP‐Glc PPase and mutant S72D and S72E. PDB codes for the WT, S72D, and S72E were 5W6J, 6V9A, and 6V96, respectively. The latter two structures were crystallized in the absence of sulfate, as described in Section 4. (a) A monomer of WT with a sulfate ligand (cyan), S72D (green), and S72E (pink) are shown overlapped. Also depicted are residues that surround the sulfate ligand and interact with it. Zoomed views of the individual structures are shown in panels (b) (WT), (c) (S72E), and (d) (S72D). Residues of the WT interacting with the sulfate are Ser72, Arg33, Arg45, and Tyr39. E) Zoomed view of the overlap of all the structures

**FIGURE 8:**
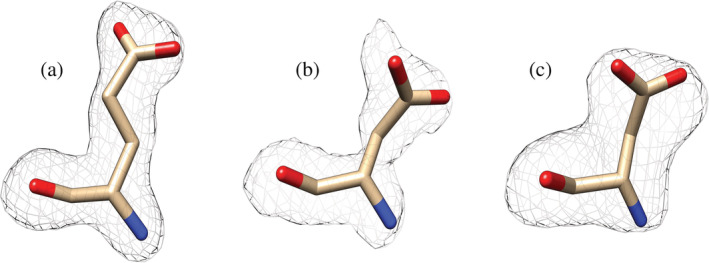
Polder maps of mutated Ser72 residue. The occurrence of the mutation has been shown by calculating the Polder (Fo‐Fc) maps of each mutant (a) S72E contoured at 4σ. (b) S72D contoured at 3σ. (c) S72D in the presence of sulfate contoured at 3.5σ

**FIGURE 9:**
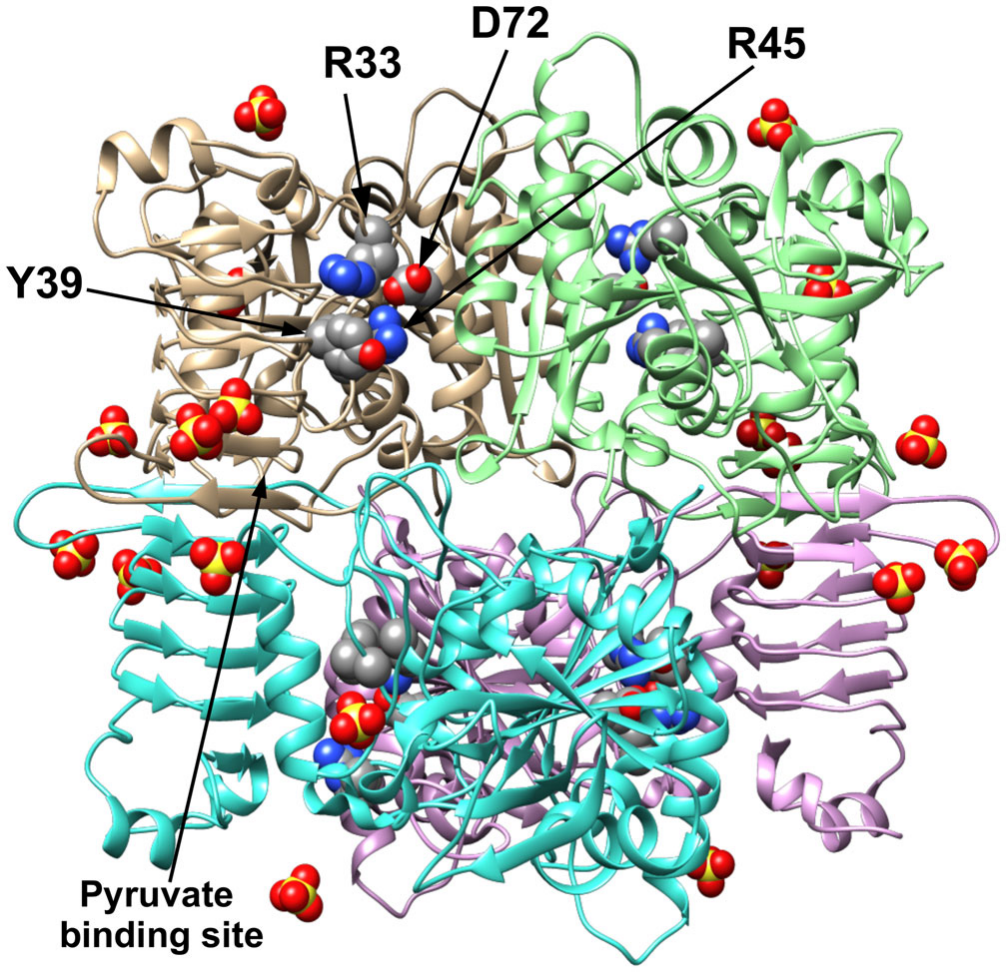
The crystal structure of ADP‐Glc PPase from *Agrobacterium tumefaciens* (mutant S72D) in the presence of sulfate (PDB code: 6V99). The figure shows the tetramer structure of the enzyme showing the Asp72, Arg33, Arg45, and Tyr39 (in the space‐filling model) colored by atom type. Conditions for crystallization were in the presence of sulfate as described in Section 4. The figure displayed well‐defined sulfate molecules in the crystal structure

The S72D and S72E mutant enzymes were crystallized in the absence of sulfate and their structures were solved with a resolution 2.04 Å and 1.8 Å, respectively (Figure 7c,d). It was evident that the side chains from Asp and Glu intrude into the space where a molecule of sulfate was in the WT (Figure 7b). Polder (Fo‐Fc) maps^22^ of the three structures obtained show that the side chains of residues in position 72 have been very well solved (Figure 8). The carboxylates from either of the side chains were located where other positively charged residues are near (Arg45 and Arg33). The bulk of these side chains and the negative charge of the carboxylate are probably the reason why a negative charge moiety from a putative ligand would not fit in this location. In fact, a crystal structure of S72D at a 2.29 Å resolution crystallized in a condition that contains sulfate in solution. However, it displays no sulfate ion in this site (Figure 9), despite other sulfate ions in other locations were observed as expected based on the WT structure.^18^ If, in the wild‐type enzyme, the phosphate group of Fru6P binds in this location, the presence of Asp or Glu in position 72 justifies the lack of binding and further activation.

## 3 DISCUSSION

Recently, the regulatory Pyr‐binding site of the *A. tumefaciens* ADP‐Glc PPase was found to be located at the C‐terminus, between two dimers of the enzyme.^18^ The binding site was near residues Ser328 and Gly329 with an important interaction of Pyr with Lys43. On the contrary, the exact description of the binding site of the other major activator (Fru6P) has remained unknown. In the *E. coli* enzyme, a synergistic effect between Pyr and sugar bisphosphate (FBP) was recently found,^20^ indicating the presence of two distinct sites. In the enzyme from *A. tumefaciens*, a similar scenario with two distinct sites is more likely than an alternative with one promiscuous regulatory site that binds both Pyr and Fru6P. For instance, it is structurally impossible to fit sugar phosphates in the very tight Pyr site.^18^ A hint for the location of another possible regulatory site is given by the presence of sulfates in the cleft between the C‐ and N‐terminal domains. These sulfates could be mimicking the phosphoryl moieties of regulatory sugar phosphates. These sulfates have been observed in the two available structures from bacterial ADP‐Glc PPase.^15^, ^16^, ^17^, ^18^ Interestingly, the crystal structure of the S subunit of the potato tuber ADP‐Glc PPase,^19^ which is activated by 3‐phosphoglycerate and inhibited by phosphate, also had sulfates in that interface. One of the sulfates in the potato tuber structure is in a position similar to where the sulfate is found in the *A. tumefaciens* enzyme. Based on modeling, it was proposed that this region could be responsible for the binding of Fru6P.^17^ A highly conserved Ser (Ser72 in *A. tumefaciens*) contacts the sulfate in all these structures (Figure 1).

To study the role of Ser72, we mutated it to Asp, Glu, Cys, Ala, and Trp, and examined the kinetic activity of the enzyme in the presence of Fru6P, Pyr, and the inhibitor AMP. All mutants lost the ability to bind the allosteric activator Fru6P (Figure 2), retaining a low basal activity. These results confirmed our hypothesis regarding the role of Ser72 in binding Fru6P. Pyr binds at the interface of the C‐terminus of a dimer, as was shown in the crystal structure of *A. tumefaciens* ADP‐Glc PPase.^18^ All mutants (S72D, S72E, S72C, and S72A) respond to Pyr (Figure 3). As mentioned above, previous mutations on Arg33 and Arg45, which occupy the same cleft as does Ser72, also maintained their sensitivity to activation by Pyr despite losing the ability to be activated by Fru6P.^14^ In addition, the thermal shift assays revealed that the substitution of Ser72 by Asp, Glu, and Ala disrupted the binding of Fru6P, while keeping the ability to bind Pyr (Figure 6). The lack of Fru6P binding explains the unchanging ATP apparent affinity of S72D and S72E in the presence of 1.5 mM Fru6P (Table S4). In addition, the presence of Fru6P does not change the saturation curves of ATP in any of the mutants (Figure 5). For this reason, the most logical explanation for the lack of activator effect is that Ser72 is directly involved in binding Fru6P rather than the alternative hypothesis that Fru6P binds, but do not trigger the activation in the mutants. In other studies in the ADP‐Glc PPase family, critical residues were determined to be involved in the signal transmission from the allosteric site to the active site, without affecting binding.^12^, ^13^, ^23^ Here, a disruption in binding of Fru6P explains the behavior of the Ser72 mutants.

Mutations at Ser72 yielded modified enzymes with limited ability to be inhibited by AMP, except for S72A (Table S3; Figure 4). This indicated that the putative hydrogen bond that Ser72 makes with the phosphoryl moiety of Fru6P is not essential to bind AMP, or that the inhibitor site is slightly different. However, the introduction of a carboxylate (Asp or Glu) that protrudes in the area where the phosphoryl group may bind, also eliminate the sensitivity to AMP inhibition. This marks a very clear distinction between mutants S72A/S72C and S72D/S72E. It is not clear why the putative interaction between Ser72 and the phosphate moiety of AMP is not important. One possible explanation is that the orientation of the phosphate when AMP is bound is not identical to the orientation of the phosphate when Fru6P binds. If that is the case, the interaction between Ser72 and the phosphate of AMP may be much weaker or not existent. The crystal structure of the AMP bound *E. coli* ADP‐Glc PPase shows that the orientation of the phosphate is not the same as the orientation that sulfate has in a different crystal (Figure S2).^15^ This actually indicates that this regulatory site has a certain flexibility to accommodate a phosphate or sulfate group, and that we cannot assume that the phosphate group from different ligands bind in the same orientation. Crystal structures of Fru6P and AMP bound to the *A. tumefaciens* ADP‐Glc PPase may provide useful information to confirm this possibility.

Our findings of mutants insensitive to inhibition could potentially have an important impact on protein engineering and biotechnology. Lack of ADP‐Glc PPase inhibition could potentially lead to a more active enzyme in vivo and, consequently, a higher production of polysaccharides. More active ADP‐Glc PPases have been used to transform plants of agricultural interest to enhance starch production.^24^, ^25^, ^26^ Using enzyme forms that are insensitive to inhibition could an interesting alternative approach even if they cannot bind the activators. A non‐inhibited basal activity could be high enough to produce high levels of polysaccharide, particularly if mutations that avoid inhibition are combined with mutations that enhance the activity in absence of activators.^10^, ^27^, ^28^

Our results indicate that a side chain with a carboxylate in position 72 blocks the binding of Fru6P and further activation, as well as the inhibition by AMP. It is possible that AMP partially shares the binding of its phosphate moiety in this location. That would be in agreement with structural data from the *E. coli* enzyme.^15^ AMP, which is a much stronger inhibitor of the *E. coli* enzyme, uses its phosphate moiety to anchor the molecule in a similar fashion. The AMP site in the *E. coli* enzyme and the *A. tumefaciens* enzyme may not be identical (for instance, the *A. tumefaciens* enzyme lacks a homologous residue to the *E. coli* Arg130 critical for interaction with the adenine base), but our results suggest that the binding of the phosphate moiety is near Ser72.

Our findings indicate that the negative charge of Asp72 or Glu72 is located at the site where the negative charges of the allosteric activator (Fru6P) would be located, thus impeding binding and activation. These mutant enzymes responded to the activation of Pyr, indicating that Pyr and Fru6P bind to different sites. The Ser72 residue seems to be far from the Pyr‐binding site that was recently found^18^ (Figure 9). Therefore, we conclude that this enzyme presents two regulatory sites that work in tandem to regulate its activity and the production of glycogen in *A. tumefaciens*. The presence of two distinct sites agrees with recent kinetic evidence that Fru6P and Pyr display synergistic effects.^29^ This indicates that there are strong structural similarities between the enzymes of this family. The enzyme from *E. coli*, despite its apparent functional differences in regulation, is proposed to also have two different regulatory sites.^11^ The presence of two distinct sites presents an excellent opportunity to evolve sensitive and dynamic responses to metabolic demands, producing enzymes with a vast regulatory diversity.^1^ This diversity in regulator specificity and synergy is essential to accommodate the metabolic needs of bacteria, experiencing vastly different environments and metabolic demands.

## 4 MATERIALS AND METHODS

### 4.1 Chemicals and supplies

Biochemicals were purchased from Sigma‐Aldrich (St. Louis, MO). Luria broth (LB) media was from USA Scientific (Ocala, FL). Malachite Green oxalate salt (MG), ammonium molybdate (AM), and tween‐20 (T20) (MG‐AM‐T20) solution were prepared as described previously.^30^ Q5‐site‐directed mutagenesis kit was from New England Biolabs (NEB; Ipswich, MA). Primers were ordered from Integrated DNA Technologies (IDT; Coralville, IA). Wizard Plus SV Miniprep and Wizard SV Gel and PCR kits were purchased from Promega (Madison, WI). Sypro Orange (20×) from Molecular Probes (Eugene, OR), and 96‐well real‐time (optical clear) PCR plate from Applied Biosystems (Waltham, MA) were used for the thermal shift assays.

### 4.2 Site‐directed mutagenesis

The wild‐type *A. tumefaciens glgC* gene coding for the ADP‐Glc PPase was subcloned in the pET28c vector as previously described.^18^ Site‐directed mutagenesis of the ADP‐Glc PPase from *A. tumefaciens* was performed with the Q5‐site‐directed mutagenesis kit as described.^18^ The primer sequences were: S72A 5′‐CAA TAT AAG GCT CAC GCC CTC ATC CGC CAC‐3′, S72D 5′‐CAA TAT AAG GCT CAC GAC CTC ATC CGC CAC‐3′, S72E 5′‐CAA TAT AAG GCT CAC GAG CTC ATC CGC CAC‐3′, S72C 5′‐CAA TAT AAG GCT CAC TGC CTC ATC CGC CAC‐3′, and S72W 5′‐GCT CAC TGG CTC ATC CGC CAC CTG C‐3′. The mutations were validated, by sending the samples for complete DNA sequencing of the gene to the University of Chicago Cancer Research center (CRC‐DNA sequencing, Chicago, IL).

### 4.3 Protein expression, purification, and crystallization

Mutant and WT plasmids were transformed into *E. coli* BL21(DE3) competent cells for expression. They were grown in 5 ml of LB with kanamycin (50 μg/ml) overnight. The cells were used to inoculate 1 L of LB media, which was shaken for ~3 h at 37°C until OD_600_ ~0.6. Then, 0.75 mM of isopropyl β‐d‐1‐thiogalactopyranoside (IPTG) was added and the culture was incubated 16 hr at 23°C. Afterward, cells were centrifuged, the supernatants discarded, and the cell pellets were re‐suspended in 15 ml of 10 mM imidazole, 200 mM NaCl, 10% (v/v) glycerol, and 50 mM HEPES (pH 7.5). The samples were sonicated, and the crude extracts were collected after centrifugation. Afterward, the His‐tagged protein was purified by fast protein liquid chromatography with a nickel column (GE Healthcare, Chicago, IL) and eluted with an imidazole gradient from 10 to 375 mM. The enzymes were concentrated by using centrifugal concentrator 30 kDa (Pall Corporation, Port Washington, NY). The mutant enzymes S72D and S72E were further purified by size exclusion chromatography (GE Healthcare HiLoad 16/600 Superdex 200 pg Column) before crystallization. The buffer for gel filtration was 50 mM HEPES (pH 7.5) and the final concentration of enzyme was adjusted to ~10 mg/ml in 50 mM HEPES (pH 7.5) and 300 mM sodium chloride buffer after concentration. The crystallization condition for either S72D or S72E was 1.2 M sodium citrate and 100 mM imidazole (pH 8). The crystallization condition for S72D (in the presence of sulfate) was 2 M lithium sulfate, 100 mM Tris (pH 8.5), and 2% (v/v) polyethylene glycol 400. The crystallization experiments were carried out using hanging drop vapor diffusion methods. Each hanging drop was formed by mixing 1 μl protein solution (10 mg/ml) with 1 μl well solution. The crystals appeared after 1 week at room temperature. To make a cryo‐protecting solution, 25% (v/v) of glycerol was added to the crystallization condition. Crystals were transfered from a hanging drop to a drop of cryo‐protecting solution on a glass coverslip before flash‐frozen in liquid nitrogen.

### 4.4 Data collection and processing

Monochromatic data sets were collected at the 19‐ID beamline‐Structural Biology Center (SBC), Advanced Photon Source (APS) at Argonne National Laboratory (ANL). Diffraction data were collected at a wavelength of 0.98 Å at 100 K using a Quantum 315r charge‐coupled device (CCD) detector from Area Detector Systems Corp. (ADSC) (Poway, CA). All collected data sets were indexed, integrated, and scaled using HKL3000^31^ or xia2^32^, ^33^, ^34^, ^35^, ^36^ in the CCP4 program suite.^34^ The best data sets were processed in space group P1 at resolutions of 2.04, 1.80, and 2.29 Å for the mutants S72D, S72E, and S72D (in presence of sulfate). Data collection statistics are in Table 1.

**TABLE 1:** Crystal structure data and refinement statistics for ADP‐Glc PPase S72E and S72D

Data processing	S72E	S72D	S72D (+sulfate)
Space group	P1	P1	P1
Cell dimension			
*α*, *β*, *γ* (°)	107.9, 101.7, 90.0	107.9, 101.6, 90.0	108.1, 101.8, 90.0
*a*, *b*, *c* (Å)	93.6, 141.2, 229.4	92.2, 141.4, 228.6	93.4, 141.6, 228.3
Resolution (Å)	50.0–1.80	77.3–2.04	72.7–2.29
Resolution at *I/σ*(*I*) = 2^a^	1.93	2.50	2.07
*I/σ*(*I*)	14.4 (1.0)^b^	4.0 (1.1)	5.6 (1.1)
*R*_merge_ ^c^ (%)	4.2 (64.7)	11.8 (71.3)	6.6 (70.1)
*R*_pim_ ^d^ (%)	4.2 (64.7)	8.3 (49.8)	6.6 (70.1)
CC_1/2_ ^e^ (%)	(68.4)	(79.5)	(65.0)
Completeness (%)	83.7 (82.0)	98.0 (94.9)	98.1 (92.0)
Multiplicity	1.7 (1.5)	3.0 (3.0)	2.0 (2.0)
No. reflections	1,475,680	1,982,497	953,035
No. unique reflections	846,330	669,633	481,239
Refinement			
*R*_work_ ^f^ */R*_free_ ^g^ (%)	19.33/22.76	23.40/27.87	20.98/25.43
No. of atoms	73,912	70,139	68,292
Protein	65,763	65,776	65,022
Ligand	392	392	680
Water	7,860	3,971	2,590
*B* factors (Å^2^)			
Protein	32.4	41.6	57.4
Solvent	40.0	39.9	50.8
r.m.s.d.^h^			
Bond lengths (Å)	0.011	0.005	0.008
Bond angles (°)	1.09	0.76	0.94
Ramachandran Plot (%)			
Favoured	95.56%	94.85%	94.67%
Allowed	3.74%	4.59%	5.08%
Outliers	0.70%	0.56%	0.25%

^a^ Provided resolution at *I*/*σ* = 2 for conventional assessment of data quality.

^b^ The values for the highest‐resolution bin are in parentheses.

^c^ *R*_merge_ = ∑|*I*_obs_ − *I*_avg_|/∑*I*_avg_.

^d^ Precision‐indicating merging *R*.

^e^ Pearson correlation coefficient of two “half” data sets.

^f^ *R*_work_ = ∑|*F*_obs_ − *F*_calc_|/∑*F*_obs_.

^g^ Five percent of the reflection data were selected at random as a test set, and only these data were used to calculate *R*_free_.

^h^ Root‐mean square deviation.

### 4.5 Structure determination, model building, and refinement

All structures were solved by molecular replacement using PHASER in the Phenix software suite.^37^ The initial search model was a single subunit of a previously published structure of ADP–Glc PPase from *A. tumefaciens* (PDB code 5W6J). The solutions of molecular replacement were then refined in Phenix through rigid‐body, isotropic, and anisotropic refinements. A twin law (h, −k, −h−l) was generated using Xtriage in Phenix and added during refinement. After each round of refinement, all models were rebuilt in Coot^38^ and refined using Phenix,^37^ and the final models were analyzed and figures were made using the programs UCSF Chimera.^39^ Final refinement statistics are presented in Table 1.

### 4.6 Enzyme assay

The activity of ADP‐Glc PPase was assayed by following the production of PPi by hydrolyzing it to Pi with inorganic pyrophosphatase as described previously.^30^ The reaction mixture (50 μl) contained 1.5 mM of ATP, 1.5 mM Glc1P, 0.2 mg/ml BSA, 7 mM MgCl_2_, 50 mM HEPES at pH 7.5, and 0.2 mg/ml pyrophosphatase. Different concentrations of activators and/or inhibitors were added as indicated. The reaction was started with the addition of 10 μl of an enzyme aliquot and the samples were incubated for 10 min at 37°C. The mixture of MG‐AM‐T20 (see Section 4.1) was added to stop the reaction and develop green color based on the amount of Pi present. At the end, the absorbance was measured at 620 nm.^30^ Standards were solutions with different concentrations of PPi treated identically as the samples. One unit (U) of enzyme activity is the amount that produces 1 μmol of PPi per minute.

### 4.7 Determination of kinetic parameters

The collected data of enzyme specific activity (U/mg) were plotted against the substrate or effector at different concentrations by using the commercial Origin 7.5 program. Experiments were run at least twice and by duplicates. We fit the data to the following modified Hill equation using a non‐linear regression algorithm (Levenberg–Marquardt):

$$V=V_{o}+\left(V_{\infty }-V_{o}\right)\;X^{n_{H}}/\left(K_{0.5}^{n_{H}}+X^{n_{H}}\right)$$

This algorithm also provided the standard errors. In this modified Hill equation, *X* is the concentration of effector ([*S*], [*A*], or [*I*] for the substrate, activator, or inhibitor, respectively), *V* is the velocity of the reaction, *V*_0_ is the velocity in absence of effector, *V*_∞_ is the velocity at saturation (infinite amount of effector). When activation or substrate saturation is analyzed, *V*_∞_ is equivalent to *V*_m_. The constant *K*_0.5_ is the concentration of effector that produces half of the velocity change between *V*_0_ and *V*_∞_, which could be expressed as *S*_0.5_, *A*_0.5,_ or *I*_0.5_ if the effector is a substrate, activator, or inhibitor, respectively. The Hill coefficient is expressed as *n*_H_.

### 4.8 Thermal shift assay

The unfolding temperature of proteins (*T*_m_) was measured in the presence and absence of ligands to evaluate their ability to bind to the protein, stabilize it, and consequently shift the *T*_m_.^40^, ^41^ Solutions of (4×) SYPRO Orange fluorescence dye (Sigma‐Aldrich), 20 mM HEPES buffer (pH 7.5), 0.02 mg/ml protein, and ligands as indicated were added in a total volume of 20 μl. The solution was placed into the wells of a 96‐well real‐time PCR plate and covered with sealing tape. The equipment used was the Step One Real‐Time PCR System^TM^ from Thermo Fisher Scientific (Waltham, MA) with its software Step One^TM^. The temperature was scanned from 25°C to 99°C and the fluorescence changes in the plate wells were recorded.^41^ The unfolding temperatures (*T*_m_) of the proteins were measured using the minimum of the negative of the first derivative of the scan fluorescence versus temperature (−*dF*/*dT*).

### 4.9 Sequence alignment of ADP‐Glc PPase protein

The sequences from different species were selected based on the reported ability to activate the ADP‐Glc PPase by Fru6P.^1^ The accession numbers were obtained from the NCBI database. Those sequences were the ones from *A. tumefaciens* (AAD03473.1), *Allochromatium vinosum* (WP_012971903.1), *Rhodobacter sphaeroides* (AAD53958.1), *Rhodomicrobium vannielii* (ADP70402.1), and *Rhodobacter capsulatus* (KQB17540.1). The alignment was performed by using the ClustalW algorithm incorporated into the BioEdit software.^42^, ^43^

## AUTHOR CONTRIBUTIONS

**Mashael A. Alghamdi:** Conceptualization (equal); formal analysis (equal); investigation (equal); methodology (equal); writing – original draft (equal); writing – review and editing (equal). **Rania A. Hussien:** Conceptualization (equal); formal analysis (equal); methodology (equal). **Yuanzhang Zheng:** Data curation (equal); formal analysis (equal); methodology (equal). **Hiral P. Patel:** Conceptualization (supporting); formal analysis (supporting); methodology (supporting). **Matías D. Asencion Diez:** Conceptualization (equal). **Alberto A. Iglesias:** Conceptualization (equal); formal analysis (equal); writing – review and editing (equal). **Dali Liu:** Conceptualization (equal); data curation (equal); formal analysis (equal); funding acquisition (equal); methodology (equal); project administration (supporting); resources (supporting); supervision (supporting); writing – review and editing (equal). **Miguel A. Ballicora:** Conceptualization (lead); formal analysis (equal); funding acquisition (lead); project administration (lead); resources (lead); supervision (lead); validation (lead); writing – original draft (equal); writing – review and editing (equal).

## Supporting information


**Table S1.** Activation by Fru6P of the *A. tumefaciens* ADP‐Glc PPase wild type and mutant enzymes.
**Table S2.** Activation by Pyruvate of the *A. tumefaciens* ADP‐Glc PPase wild type and mutant enzymes.
**Table S3.** Inhibition by AMP of the *A. tumefaciens* ADP‐Glc PPase wild type and mutant enzymes.
**Table S4.** ATP saturation curve parameters of *A. tumefaciens* ADP‐Glc PPase wild type and mutant enzymes in the presence and absence of Fru6P.
**Table S5.** Thermal shift assay of the *A. tumefaciens* ADP‐Glc PPase wild type and mutant enzymes in the presence of substrates.
**Table S6.** Thermal shift assay of the *A. tumefaciens* ADP‐Glc PPase wild type and mutant enzymes in the absence of substrates.
**Figure S1.** Effect of activators on the thermal stability of the *A. tumefaciens* ADP‐Glc PPase (in absence of ATP).
**Figure S2.** Overlap of the *E. coli* ADP‐Glc PPase with AMP and sulfate bound.

## Data Availability

The data that support the findings of this study are available from the corresponding author upon reasonable request.
