# Supplementary material for: Site‐directed mutagenesis of Serine‐72 reveals the location of the fructose 6‐phosphate regulatory site of the Agrobacterium tumefaciens ADP‐glucose pyrophosphorylase
Source: Protein Sci. 2022 Jun 26;31(7):e4376. doi: 10.1002/pro.4376 (PMC9234290; doi:10.1002/pro.4376)
Supplement: Supplementary file 1 — Table S1. Activation by Fru6P of the A. tumefaciens ADP‐Glc PPase wild type and mutant enzymes. Table S2. Activation by Pyruvate of the A. tumefaciens ADP‐Glc PPase wild type and mutant enzymes. Table S3. Inhibition by AMP of the A. tumefaciens ADP‐Glc PPase wild type and mutant enzymes. Table S4. ATP saturation curve parameters of A. tumefaciens ADP‐Glc PPase wild type and mutant enzymes in the presence and absence of Fru6P. Table S5. Thermal shift assay of the A. tumefaciens ADP‐Glc PPase wild type and mutant enzymes in the presence of substrates. Table S6. Thermal shift assay of the A. tumefaciens ADP‐Glc PPase wild type and mutant enzymes in the absence of substrates. Figure S1. Effect of activators on the thermal stability of the A. tumefaciens ADP‐Glc PPase (in absence of ATP). Figure S2. Overlap of the E. coli ADP‐Glc PPase with AMP and sulfate bound. [file PRO-31-e4376-s001.pdf]

Supplemental Information

Table S1

Activation by Fru6P of the *A. tumefaciens* ADP-Glc PPase wild type and mutant enzymes

Enzyme [a]	Fru6P				
	V_0 (U/mg)	V_m (U/mg)	$A_{0.5}$ (mM)	n_H	Activation (V_m/V_o) -fold
WT	8.2 ± 2.1	98.5 ± 1.5	0.10 ± 0.01	1.9 ± 0.1	12
S72D	6.7 ± 0.9	8.3 ± 0.4	N/A [b]	N/A [b]	1.2
S72E	5.5 ± 0.2	8.9 ± 0.4	N/A [b]	N/A [b]	1.6
S72A	7.9 ± 0.3	7.8 ± 0.3	N/A [b]	N/A [b]	1.0
S72C	4.6 ± 0.2	9.1 ± 1.4	N/A [b]	N/A [b]	2.0
S72W	0.6 ± 0.1	1.3 ± 0.5	N/A [b]	N/A [b]	2.1

[a] Assays were performed as described in Materials and Methods.

[b] Activation was not significant enough to determine the parameter with precision.

Table S2

Activation by Pyruvate of the *A. tumefaciens* ADP-Glc PPase wild type and mutant enzymes

Enzyme [a]	Pyruvate				
	V_0 (U/mg)	V_m (U/mg)	$A_{0.5}$ (mM)	n_H	Activation (V_m/V_o) -fold
WT	7.9 ± 5.6	79.2 ± 1.6	0.057 ± 0.007	1.8 ± 0.3	10.1
S72D	6.6 ± 0.2	38.6 ± 0.5	0.018 ± 0.001	2.6 ± 0.4	5.8
S72E	5.8 ± 0.7	39.5 ± 0.7	0.064 ± 0.004	1.8 ± 0.2	6.8
S72A	8.1 ± 4.3	49.1 ± 0.2	0.083 ± 0.012	1.5 ± 0.2	6.1
S72C	4.0 ± 0.9	18.8 ± 1.8	0.330 ± 0.080	1.0 ± 0.3	4.7
S72W	0.9 ± 0.1	2.3 ± 0.3	N/A [b]	N/A [b]	2.6

[a] Assays were performed as described in Materials and Methods.

[b] Activation was not significant enough to determine the parameter.

Table S3

Inhibition by AMP of the *A. tumefaciens* ADP-Glc PPase wild type and mutant enzymes

Enzyme [a]	AMP				
	V_0 (U/mg)	V_∞ (U/mg)	$I_{0.5}$ (mM)	n_H	V_∞/V_0 (%)
WT	57.3 ± 6.9	3.5 ± 1.3	0.40 ± 0.08	1.57 ± 0.31	6.1
S72D	5.33 ± 0.15	1.1 ± 1.7	> 5 [b]	N/A [c]	20 [b]
S72E	5.35 ± 0.14	1.46 ± 0.40	> 5 [b]	N/A [c]	27 [b]
S72A	7.90 ± 0.80	0.60 ± 0.50	0.6 ± 0.1	1.0 ± 0.2	7.6
S72C	4.62 ± 0.23	0.64 ± 0.28	> 5 [b]	N/A [c]	13.9 [b]
S72W	0.42 ± 0.01	0.30 ± 0.02	N/A [c]	N/A [c]	71 [b]

[a] Assays were performed as described in Materials and Methods, with a concentration of 0.1 mM Fru6P.

[b] Inhibition was observed at higher concentrations, but it is uncertain whether it was specific.

[c] Inhibition was not significant enough to determine the parameter with accuracy.

Table S4

ATP saturation curve parameters of *A. tumefaciens* ADP-Glc PPase wild type and mutant enzymes in the presence and absence of Fru6P

Enzyme [a]	Fru6P (mM)	ATP $S_{0.5}$ (mM)	ATP n_H	ATP V_m (mM)
WT	0	0.66 ± 0.18	1.31 ± 0.25	11.2 ± 1.6
	1.5	0.17 ± 0.01	1.42 ± 0.05	76.2 ± 0.6
S72D	0	0.67 ± 0.15	1.20 ± 0.23	6.8 ± 0.7
	1.5	0.62 ± 0.09	1.48 ± 0.26	9.1 ± 0.7
S72E	0	0.78 ± 0.13	1.39 ± 0.25	6.5 ± 0.5
	1.5	0.78 ± 0.06	1.82 ± 0.25	6.4 ± 0.3
S72A	0	0.37 ± 0.04	2.19 ± 0.46	10.1 ± 0.5
	1.5	0.57 ± 0.13	1.53 ± 0.35	11.5 ± 1.6
S72C	0	1.13 ± 0.22	1.48 ± 0.29	7.3 ± 0.8
	1.5	2.14 ± 0.60	1.40 ± 0.23	10.7 ± 1.9
S72W	0	3.04 ± 0.82	0.67 ± 0.17	1.1 ± 0.5
	1.5	0.55 ± 0.28	1.27 ± 0.41	1.9 ± 0.6

[a] Assays were performed and parameters were calculated as described in Materials and Methods. Curves were performed in presence and absence of 1.5 mM Fru6P as indicated.

Table S5

Thermal shift assay of the *A. tumefaciens* ADP-Glc PPase wild type and mutant enzymes in presence of ATP and $MgCl_2$

	T_m (℃)		
	Control	+Fru6P	+Pyr
WT	54.82 ± 0.13	61.78 ± 0.13	57.53 ± 0.93
S72A	51.52 ± 0.20	51.91 ± 0.16	57.27 ± 0.50
S72D	63.53 ± 0.34	63.93 ± 0.12	66.2 ± 0.24
S72E	66.15 ± 0.23	65.10 ± 0.47	67.35 ± 0.66

[a] Unfolding temperatures (T_m) were measured as indicated in Materials and Methods with the additional presence of 1 mM ATP and 1 mM $MgCl_2$ in all wells. Thermal shift assays were performed for the WT and the mutants in presence and absence (control) of Fru6P (1 mM) and Pyr (1 mM)

Table S6

Thermal shift assay of the *A. tumefaciens* ADP-Glc PPase wild type and mutant enzymes

	T_m (°C)		
	Control	+Fru6P	+Pyr
WT	56.72 ± 0.32	60.01 ± 0.18	60.10 ± 0.19
S72A	53.95 ± 0.28	53.78 ± 0.28	57.70 ± 0.25
S72D	63.25 ± 0.37	63.78 ± 0.37	65.17 ± 0.19
S72E	65.98 ± 0.18	65.33 ± 0.18	66.01± 0.50

[a] Unfolding temperatures (T_m) were measured as indicated in Materials and Methods. No ATP or $MgCl_2$ were present. Thermal shift assays for the WT and the mutants in presence and absence (control) of Fru6P (1 mM) and Pyr (1 mM)

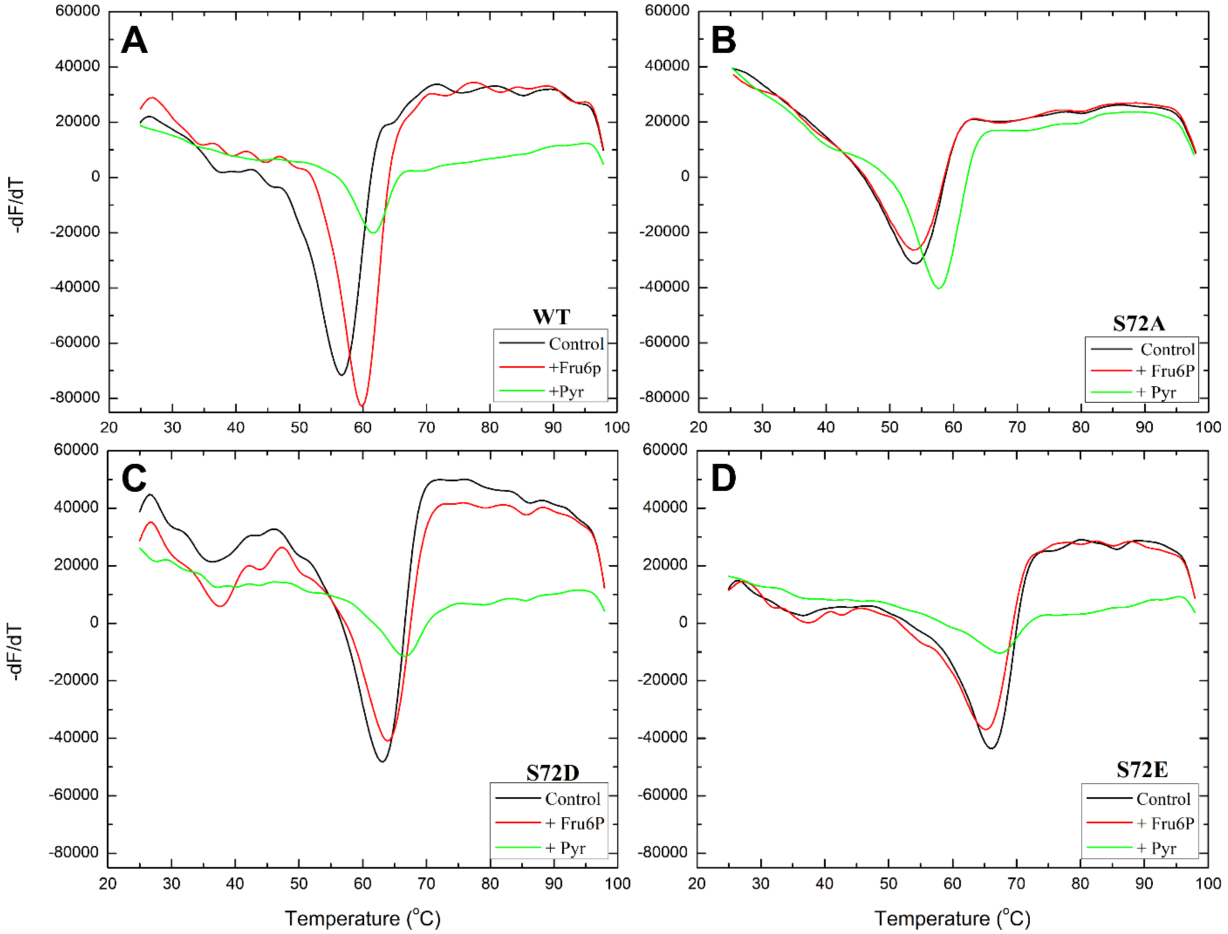

Figure S1. Effect of activators on the thermal stability of the *A. tumefaciens* ADP-Glc PPase (in absence of ATP). Thermal shift assays for the WT and the mutants in presence and absence (control) of Fru6P (1 mM) and Pyr (1 mM) were performed as described in Materials and Methods. We depicted in panels A, B, C, and D, the wild type and mutants S72A, S72D, and S72E, respectively.

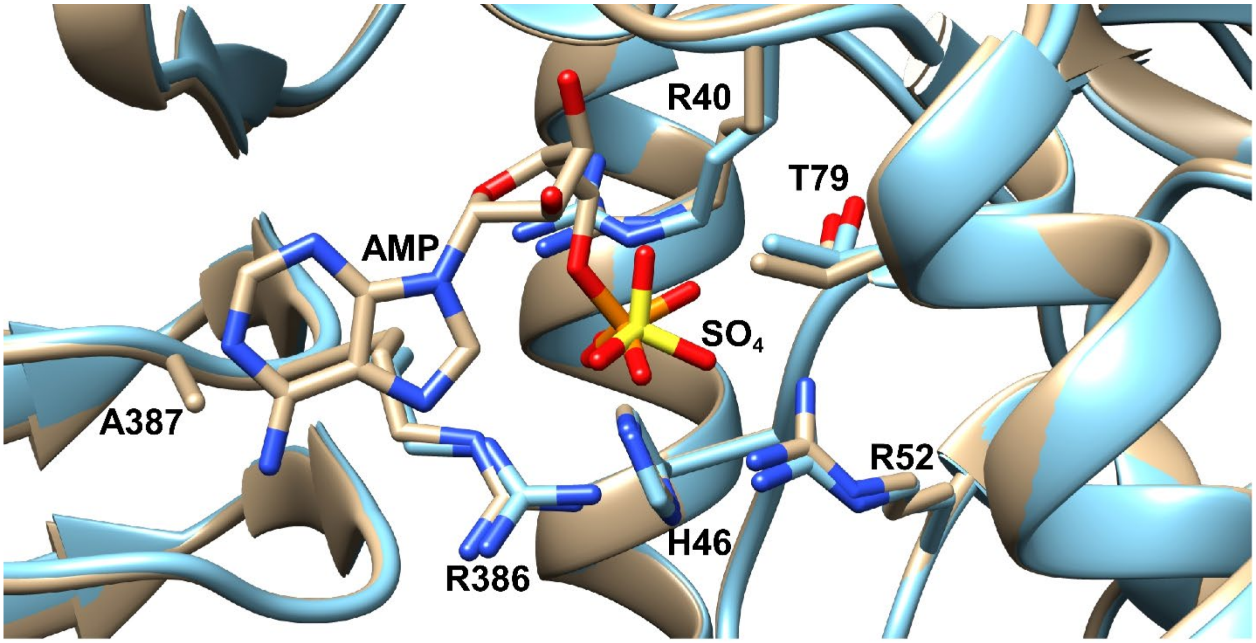

Figure S2. Overlap of the *E. coli* ADP-Glc PPase with AMP and sulfate bound. The tan colored structure is the AMP bound structure (PDB: 5L6V) and the light blue is the one with sulfate in the same location (PDB: 5L6S). Sulfate is in yellow, and the phosphate moiety of AMP is in orange.
